# A Two-Year Retrospective Analysis of the Clinical Outcomes of Immediate Submuscular Breast Reconstructions With Native® Acellular Dermal Matrix

**DOI:** 10.7759/cureus.41343

**Published:** 2023-07-04

**Authors:** Larissa L Martins, Rui F Barbosa, Frederico C Guerreiro, Carolina Andresen, Miguel J Pereira, Carlos J Pinho, Marco A Rebelo, Matilde M Ribeiro

**Affiliations:** 1 Department of Plastic, Reconstructive, Craniomaxillofacial and Hand Surgery and Microsurgery Unit, Centro Hospitalar Vila Nova de Gaia/Espinho, Vila Nova de Gaia, PRT; 2 Department of Plastic Surgery, Hospital da Prelada, Porto, PRT; 3 Department of Plastic and Reconstructive Surgery, Instituto Português de Oncologia do Porto Francisco Gentil, EPE, Porto, PRT; 4 Department of Reconstructive and Maxillofacial Plastic Surgery, Centro Hospitalar de Lisboa Ocidental, Lisbon, PRT

**Keywords:** breast reconstruction, oncoplastic surgery, breast neoplasms, breast implants, acellular dermis

## Abstract

Background: Implant-based submuscular breast reconstruction (SBR) can be performed with the aid of acellular dermal matrices (ADM) for implant coverage on their inferolateral pole, aiming at providing a biological interface for hiding the implant and therefore reducing the risk of complications. The purpose of this study is to assess the long-term post-operative outcomes obtained using the SBR-specific Native® ADM (DECO med s.r.l., Marcon, Venice, Italy).

Methods: All cases of Native®-assisted immediate SBR performed at our institution between October 2016 and March 2020 were retrospectively analysed. Demographic and surgical data were collected, and post-operative outcomes, including minor and major complications, were evaluated. Particular attention was paid to complications emerging before and after patient discharge. Dependence analyses were performed to uncover statistically significant relationships between risk factors and reconstructive outcomes.

Results: Data on 100 patients were collected, for a total of 128 breasts. The mean age of the cohort was 49.5 years, the mean BMI was 23.4 kg/m2, and the mean follow-up was 24 months. Out of this, 14.1% of patients received pre-operative radiotherapy, while 16.4% underwent post-mastectomy radiotherapy. Breasts appeared to develop short-term minor complications more likely during hospitalisation (11.7% vs. 7.8%), while short-term major complications occurred more often after discharge (7.8% vs. 15.6%). The most frequent long-term complications were capsular contracture and contour defects (both 9.4%). Risk factors that showed a statistically significant relationship with complications were pre- and post-mastectomy radiotherapy and post-operative chemotherapy.

Conclusions: The retrospective analysis showed results in line with clinical outcomes reported in the literature for the same reconstructive technique. The use of Native® ADM in SBR is safe and effective in the long term.

## Introduction

Immediate breast reconstruction (IBR) following mastectomy is an oncologically safe procedure that also improves patients' psychosocial health. Therefore, nowadays, surgeons in over one-third of European countries perform IBR in more than 20% of mastectomy cases [1].

Submuscular breast reconstruction (SBR) is a consolidated reconstructive practice that has seen technical evolution over the years [2,3]. In immediate SBR, the implant is placed between the pectoralis major muscle and the chest wall. It can be completely inserted in the sub-muscular pocket, or it can be covered partially by the pectoralis major muscle and partially by an acellular dermal matrix (ADM) sling on the lower pole in a dual-plane procedure in cases of bigger implant dimensions [4,5]. The use of ADMs has gained more and more popularity in IBR since their advent in 2005 because they made it possible to achieve a better inframammary fold definition and allowed the use of implants with more adequate size and projection. This technique improved overall aesthetic results while also providing a protective effect against capsular contracture by shielding the subcutaneous tissue from the presence of the synthetic implant [2,6,7].

Biological matrices are bioactive scaffolds that, for intrinsic structural and chemical properties, can interact with the natural body’s healing process and support tissue regeneration after surgical damage [8]. Their safety and efficacy in breast reconstruction have been widely studied and proven; similar reconstructive outcomes and complication rates were observed between patients reconstructed with ADM and with an autologous dermal flap [9]. Research in the bioengineering field has also highlighted the importance of having biomaterials that are tissue-specific for the implant site in order to obtain regenerative healing not only on the functional but also on the anatomical side [10,11]. Native® ADM (DECO med s.r.l., Marcon, Venice, Italy) is the only biomaterial specifically designed for subpectoral breast reconstruction, and literature data confirm its effectiveness in both clinical and aesthetic terms [9,12-14]. However, the long-term results of a larger patient cohort have not yet been reported in the scientific literature. This study focuses on the long-term clinical outcomes of Native®-assisted SBR in a large cohort of patients.

## Materials and methods

This retrospective study was conducted on 100 patients (128 breasts) who underwent direct-to-implant submuscular breast reconstruction with an ADM sling between October 2016 and March 2020 at the Instituto Português de Oncologia do Porto, Porto, Portugal.

Inclusion criteria included patients of the female gender, those aged above 18 years, and patients who underwent direct-to-implant submuscular breast reconstruction with an ADM sling with Native® biological matrix. Exclusion criteria included patients who underwent other types of breast reconstruction, submuscular reconstruction with tissue expanders, or had less than one month of follow-up. Patients with advanced disease at the initial diagnosis were also excluded from the study, as it represents a contraindication for immediate reconstruction.

Immediately after skin and nipple-sparing mastectomy, the pectoralis major muscle was inferolaterally detached from the rib cage to create the pocket. All reconstructions were performed using Native® biological matrix as lower/lateral-pole coverage of the breast implants. The ADM was used as per the manufacturer's instructions. Briefly, Native® was removed from its envelope, ensuring sterility was maintained, and was submerged for hydration in a sterile saline solution at room temperature. After five minutes, the ADM was pliable and ready for implantation. Native® is a rectangular collagen sheet available in three different sizes (15x8 cm, 18x10 cm, and 22x12 cm). The most appropriate ADM size was chosen considering the dimension of the implant and stitched using absorbable sutures. In cases of inframammary access, Native® was fixed with separate stitches to the edge of the detached muscle and then to the inframammary fold following implant placement, while in cases of periareolar access, the ADM was first fixed to the inframammary fold and then to the edge of the detached muscle so as to complete the pocket for implant allocation. The matrix can be shaped to fit the prosthesis’ curved silhouette. Two drains were inserted, one between the implant and the ADM and one between the ADM and the subcutaneous tissue. Drains were removed when the output presented a decreasing trend and decreased below 30 ccs for 24 hours. The incision was closed after wound edge excision, and a conforming dressing was applied. A sports bra was recommended starting five days after surgery. Patients were administered intravenous antibiotic prophylaxis at induction, either with cefazolin, ciprofloxacin, or a combination of amoxicillin and clavulanate, and they continued with oral antibiotics (amoxicillin and clavulanate, or if allergic, ciprofloxacin) for one week.

Demographic data such as age, BMI, comorbidities, smoking habits, previous surgeries, and previous therapies were recorded. Patients who had stopped smoking at least six months pre-operatively were considered non-smokers. Complications such as seroma, dehiscence, necrosis, hematoma, infection, and capsular contracture were recorded. Depending on the timing of their onset, they were classified as before or after discharge from the hospital. Reinterventions and implant removals were recorded as well. Complications that required reintervention or led to reconstructive failure were considered major.

The data were analysed and presented using basic descriptive statistical tools. For descriptive analysis, relative frequencies were calculated, while for continuous variables, their respective means and standard deviations were obtained. Independent group comparison tests were conducted whenever categorical variables were compared with continuous variables (independent t-test), and association tests were performed when analysing categorical variables among themselves (chi-square test or Fisher's exact test).

## Results

A hundred patients were retrospectively analysed for the study, for a total of 128 breasts, all operated at our institution between October 2016 and March 2020. The mean age was 45.9 years, and the mean BMI was 23.4 kg/m2; 64% of patients were non-smokers, 6% were previous smokers, and 13% were smokers (for 17%, their smoking habit was unknown). The prevalence of comorbidities was 9% for hypertension, 3% for diabetes, 3% for autoimmune diseases, 3% for pulmonary diseases, 3% for thyroid diseases, and 2% for cardiac pathologies. Thirty-eight patients underwent risk-reducing surgeries, while 62 had therapeutic mastectomies. Post-operative chemotherapy was administered to 37 patients, while 14 received the chemotherapy treatment pre-operatively; 51 patients received hormonal therapy; 18 patients had previous radiation therapy (RT); and 21 patients in the study received post-operative RT. Patients in our institution remain in the hospital following surgery until drains are removed, with a mean hospital stay of 13 days for the present collection. The mean follow-up of the patient cohort was 24 months. Demographic data and surgical details of the participants are reported in Table 1.

**Table 1:** The participants' demographic and surgical data

Demographic details	No. (± S.D./percentage)
Number of patients	100
Number of breasts	128
Mean age (in years)	45.9 (± 9.1)
Mean BMI (kg/m^2^)	23.4 (±3.3)
Mean follow-up (months)	24 (± 15)
Hospitalisation (number of days)	13 (± 11)
Smoking status (per patient)	
Active smokers	13 (13)
Non-smokers	64 (64)
Previous smokers	6 (6)
Unknown	17 (17)
Comorbidities	
Diabetes	3 (3)
Hypertension	9 (9)
Cardiac pathologies	2 (2)
Pulmonary disease	3 (3)
Thyroid disease	3 (3)
Autoimmune disease	3 (3)
Previous breast surgery	27 (21.1)
Congenital risk (mutations, familiarity)	29 (29)
Surgical details	No. (± S.D./percentage)
Bilateral mastectomies	32 (50)
Unilateral mastectomies	64 (50)
Therapeutic surgeries	90 (70.3)
Prophylactic surgeries	38 (3)
Nipple preservation (per breast)	
Yes	59 (46.1)
No	48 (37.5)
Graft	13 (1)
Mean gland weight (g)	354 (± 202)
Mean implant volume (cc)	383 (± 103)
Axillary lymph node biopsy	62 (48.4)
Mean time of drains permanence (days)	9 (± 4)
Therapeutic treatment (per patient/breast)	
Pre-op chemotherapy	14 (14)
Pre-op radiotherapy	16 (12.5)
Post-op chemotherapy	37 (37)
Post-op radiotherapy	21 (16.4)
Hormone therapy	51 (51)

The purpose of the surgery, when the surgery was bilateral, is specified in Table 2. 

**Table 2:** The aim of the surgery when the patient was referred for bilateral surgery

Bilateral mastectomy aim	N (%)
Therapeutic: both breasts	5 (18)
Prophylactic: both breasts	8 (29)
1 breast therapeutic + 1 breast prophylactic	15 (54)

The data on total complications (calculated per breast) are reported in Table 3.

**Table 3:** Secondary interventions in the long term (on the 103 reconstructions that did not undergo implant loss due to early complications)

Secondary operation	N (%)
Revision with prosthesis removal/substitution	9 (8.7%)
Lipofilling	22 (21.4%)

Considering minor complications, which saw resolution in outpatient settings (or for which reintervention was not required), dehiscence was the most frequent, observed in 13.3% of reconstructions. Seroma was at 2.3%, and infection was at 1.6%. No haematomas were observed. Major complications that required surgical intervention were dehiscence (14.1%), necrosis (5.5%), infection (4.7%), seroma, and haematoma (both 1.6%). Implant loss was set at 19.5%.

Considering the timing of complication occurrence, it emerged that minor complications had slightly higher chances of developing during hospitalisation (9.4% vs. 7.0%). Dehiscence was the most frequent complication in both settings (7.8% during hospitalisation, 5.5% after discharge). Minor infections occurred only before discharge (1.6%), while seroma occurrences occurred more often after discharge (0.8% vs. 1.6%).

The frequency of major complications was twofold after discharge from the hospital (7.8% vs. 15.6%). The most frequent one emerging during hospitalisation was necrosis (5.5%), followed by infection (1.6%) and haematoma (0.8%). No seroma formed during this early post-operative period. After discharge, the most frequent complication was dehiscence (14.1%), with infection, seroma, and haematoma reported less frequently (3.1%, 1.6%, and 0.8%, respectively). No necrosis was recorded. Implant loss was documented more frequently after discharge (5.5% vs. 14.1%). All these complications were considered short-term because they were strictly linked to the surgical intervention.

Long-term sequelae were analysed separately and are reported in Table 4.

**Table 4:** Total short-term complications are divided into minor and major complications, with subdivisions in complications occurring before and after discharge from the hospital.

Complications	Total	Before discharge	After discharge
Any minor complication	21 (16.4%)	12 (9.4%)	9 (7.0%)
Haematoma	0 (0.0%)	0 (0.0%)	0 (0.0%)
Infection	2 (1.6%)	2 (1.6%)	0 (0.0%)
Seroma	3 (2.3%)	1 (0.8%)	2 (1.6%)
Dehiscence	17 (13.3%)	10 (7.8%)	7 (5.5%)
Any major complication	30 (23.4%)	10 (7.8%)	20 (15.6%)
Haematoma	2 (1.6%)	1 (0.8%)	1 (0.8%)
Necrosis	7 (5.5%)	7 (5.5%)	0 (0.0%)
Infection	6 (4.7%)	2 (1.6%)	4 (3.1%)
Seroma	2 (1.6%)	0 (0.0%)	2 (1.6%)
Dehiscence	18 (14.1%)	0 (0.0%)	18 (14.1%)
Implant loss	25 (19.5%)	7 (5.5%)	18 (14.1%)

In the long term, nine breasts (9.4%) required reoperation with prosthesis removal or exchange, seven of them due to capsular contracture; 22 (21.4%) were indicated for lipofilling, two of them for mild contracture.

An analysis of the features of surgical and therapeutic procedures (those that can be considered risk factors with an influence on the reconstructive outcomes) has been carried out (Tables 5-10).

**Table 5:** Dependency testing of total complications and clinical variables RR: relative risk; RT: radiotherapy. 𝝌²: chi-square test for independence; F: Fisher’s exact test.

Clinical variable	Total complications		RR	p-value
Smoking status	Yes	No		
Smokers/previous smokers	10	17	1.039	.893 (𝝌²)
Non-smokers/unknown	36	65		
Radiotherapy (RT)				
Previous RT	9	7	1.703	.070 (𝝌²)
No previous RT	37	75		

**Table 6:** Dependency testing of minor complications and radiotherapy RR = relative risk; RT = radiotherapy. F = Fisher’s exact test.

Clinical variable	Minor complications		RR	p-value
Radiotherapy	Yes	No		
Previous RT	4	12	1.750	.276 (F)
No previous RT	19	96		

**Table 7:** Dependency testing of capsular contracture and clinical variables RR: relative risk; CT: chemotherapy; PMRT: post-operative radiotherapy; F: Fisher’s exact test

Clinical variable	Capsular contracture		RR	p-value
CT	Yes	No		
Post-operative CT	10	32	10.23	< .05 (F)
No post-operative CT	2	84		
PMRT				
PMRT	11	10	28.02	< .05
No PMRT	2	105		
Smoking status				
Smokers/previous smokers	5	22	2.672	.128 (F)
Non-smokers/unknown	7	94		

**Table 8:** Dependency testing of major complications and radiotherapy RR: relative risk; RT: radiotherapy; F: Fisher’s exact test

Radiotherapy	Major complications		RR	p-value
	Yes	No		
Previous RT	6	10	1.750	.205 (F)
No previous RT	24	88		

**Table 9:** Dependency testing of implant loss and clinical variables RR: relative risk; RT: radiotherapy; NAC: nipple-areola complex; 𝝌²: chi-square test for independence; F: Fisher’s exact test

Clinical variable	Implant loss		RR	p-value
Radiotherapy	Yes	No		
Previous RT	6	10	2.211	.085 (F)
No previous RT	19	93		
Incision				
IF incision	6	16	1.522	.375 (F)
No IF incision	19	87		
Smoking status				
Smokers/previous smokers	5	22	0.935	.881 (𝝌²)
Non-smokers/unknown	20	81		
NAC preservation				
NAC preservation	10	51	0.732	.393 (𝝌²)
No NAC preservation	15	52		
Skin necrosis				
Necrosis	7	0	6.722	<.05
No necrosis	18	103		

**Table 10:** Dependency testing of major and continuous clinical variables BMI: body mass index; t: t-test

Clinical variable	Major complications		p-value
	Yes	No	
Implant size	386.17 cc (±94.7)	379.69 cc (±105.32)	.764 (t)
BMI	24.02 kg/m^2^ (±3.14)	23.31 kg/m^2^ (±3.4)	.308 (t)
Age	41,7 years (±7.10)	46,22 years (±9.34)	.016 (t)

Considering radiation therapy, 16 breasts had this treatment before surgery with a higher risk of short-term minor complications, but it did not result in a statistically significant difference. The same can be affirmed for major early complications and for implant loss (Tables 8-9).

A statistically significant dependence was observed between adjuvant chemotherapy and capsular contracture, with chemotreated patients having a higher risk of this complication (p < .05). Capsular contracture was observed to be dependent on post-operative radiotherapy as well (p < .05). No other significant dependence was found between the onset of major complications and categorical parameters.

Implants slightly larger had been used in patients that developed major complications, yet the difference was not significant. Instead, age was found to be significantly lower in that group, with patients developing complications being nearly five years younger (41.7 years vs. 46.22 years).

## Discussion

As techniques and technologies evolve, treatment for breast cancer is shifting towards personalised medicine [15]. Advancements in both mastectomy and reconstructive surgery have finally allowed safe and effective implant placement, yet with the present variety of operative options, choosing the best procedure requires a thorough understanding of the benefits and drawbacks of different techniques with regard to each specific patient and procedure [16].

In this retrospective study, we analyse a four-year data collection of ADM-assisted immediate submuscular breast reconstructions, all performed with Native® dermal matrices. Remarkably, decellularised dermis-derived materials have been a substantial breakthrough in the development of new reconstructive surgeries, acting as regenerative scaffolds and preventing prostheses-induced foreign body responses [11,17,18]. As a result, ADM-assisted prepectoral breast reconstruction (PPBR) is certainly one of the most successful achievements of the present day and is even regarded as a possible new gold standard [19]. Nonetheless, prepectoral procedures are not suitable for all women and are based on strict selection criteria. Several patient characteristics, such as a history of significant comorbidities, radiation, or active smoking, portend a higher risk of complications with prepectoral reconstruction, in which case subpectoral implant placement may be a safer option [15,20].

In this scenario, ADM-assisted dual plane procedures have been credited with enabling one-stage surgeries long before the advent of the prepectoral technique, reducing the incidence of deleterious fibrotic reactions, and extending breast reconstruction to larger implants [7,15]. As numerous pieces of histological evidence have been proving ADM to prevent fibrotic-inflammatory responses [21,22], both short- and long-term analyses have since reported a low incidence of fibrotic complications when reconstructions are performed with the use of these materials [4,6,7,23]. These observations have also been confirmed in comparative studies that found a significantly lower incidence of capsular contracture in acellular dermal matrix-assisted reconstructions versus standard reconstructions [24].

However, although the submuscular ADM-assisted technique is now extensively documented, the choice of reconstruction materials should always be evaluated, updating clinical practice with increasingly extensive follow-ups. Furthermore, in the current broad panorama of reconstruction-declined devices, different materials may lead to different outcomes: source, preparation, and thickness of acellular dermal matrices may influence the development of post-operative complications [25]. Therefore, each device should be investigated individually and independently.

In our series, we used a 0.6-mm-thick porcine dermal matrix as the lower pole coverage of the prosthetic implant. Native® ADM has been used in SBR since 2014. The first article reported low complications and natural aesthetic outcomes of the reconstructed breasts, along with complete matrix integration two months after surgery [12]. Additional and more recent studies show how, on large cohorts, Native®-assisted SBR has favourable outcomes not only when patients present risk factors (such as high BMI, old age, or tobacco use) [14], but also when this reconstructive technique is compared to autologous breast reconstruction with an inferior dermal flap, evidencing how Native® can compensate for the lack of autologous tissue [9].

Early complications are closely related to the surgical act itself, which can produce favourable or unfavourable outcomes depending on tissue quality and the procedure. One major risk factor for tissue viability is previous radiation treatment on the tissues involved in the surgery, and we decided to test this factor. It resulted in minor complications, and complications, in general, were higher in the previously radiotreated group, though their dependency was not result-significant. With similar trends, this relationship was not confirmed with major complications or implant loss as well, indicating that surgical accuracy can stem detrimental processes in higher-risk patients. Concerning PMRT, as one ascertained risk factor for capsular contracture (CC), it significantly influenced long-term outcomes in our court as well, resulting in a 25-fold risk of contracture development with respect to non-irradiated cases. Accordingly, despite the proven protective effect of dermal matrices, PMRT on subpectoral reconstructions has pro-fibrotic effects on the pectoralis major muscle, which will tend to contract and affect the final aesthetics of the reconstructed breast in the long term.

Exploring the influence of other indispensable oncological treatments, post-operative chemotherapy has shown no dependence relationship with the general development of early complications nor with the occurrence of implant loss due to post-discharge complications (considering that chemotherapy is not administered any earlier), yet capsular contracture resulted depending on post-operative chemotherapy administration. However, it must be considered that PMRT is also linked to the administration of adjuvant chemotherapy, so the effect on CC shall not be attributed to the latter on a causative basis.

In our experience, seroma as a minor and major complication occurred in a limited number of cases (five reconstructions, 3.9%); in fact, it is our practice to place two drains in the breast pocket and to remove drains only when, in a decreasing trend, their output drops below 30 cc/die. This affects hospital stay length, which is about 13 days in our series, yet it must be taken into account that some patients also received asymmetrical reconstructions where the contralateral breast was reconstructed with an implant and latissimus dorsi flap, thus having a longer drainage period.

Implant loss, as a final measure of a successful reconstruction, resulted in a rate as high as 19.5% in our series. Nevertheless, most cases of implant loss were caused by an early event of skin flap necrosis, with a significant dependence between the two (p < .05). This highlights the paramount importance of the demolition part of the procedure, which cannot overlook oncological safety and yet bears the responsibility of coping with the necessities of immediate reconstruction in appropriate cases, i.e., preserving a viable subcutaneous layer that is vascularly autonomous and able to accept the prosthetic implant.

The retrospective analysis and the single-centre nature of the data collection are the limitations of this work. In addition, general information gaps on risk factors such as the type of incision or smoking habits were assessed. The study population was found to be highly heterogeneous, with patient selection criteria generally more inclusive than standard direct-to-implant guidelines regarding mastectomy flap thickness and comorbidities, with a likely negative impact on final outcomes. Lastly, patient satisfaction assessment tools were not used in our aesthetic data, and these ratings may provide a more accurate aesthetic assessment of the final reconstructed breast.

## Conclusions

Exploring the long-term impact of specific biomaterials on breast reconstruction will contribute to the growing body of literature and may also play a key role in predicting clinical outcomes. Our retrospective study focuses on the long-term results of immediate subpectoral breast reconstructions performed using Native® ADM as implant coverage on the inferolateral pole. Our results are in line with complication ranges published in the literature and confirm that Native®-assisted SBR is a safe and effective procedure, even in longer follow-ups.
